# What tool do undergraduate pharmacy students prefer when grading systematic review evidence: AMSTAR‐2 or ROBIS?

**DOI:** 10.1002/cesm.12023

**Published:** 2023-08-09

**Authors:** Shaun W. H. Lee

**Affiliations:** ^1^ School of Pharmacy Monash University Malaysia Subang Selangor Malaysia; ^2^ School of Pharmacy Taylor's University Subang Selangor Malaysia; ^3^ Center for Global Health University of Pennsylvania Philadelphia Pennsylvania USA

**Keywords:** AMSTAR‐2, grading, risk of bias, ROBIS, systematic reviews, teaching

## Abstract

**Introduction:**

While systematic reviews (SRs) are considered the highest form of evidence in the hierarchy, the quality and standard of reviews varies. Two quality assessment tools have been developed to assess the variation in such standards. This study compared the preference, validity, reliability, and applicability of using A Measurement Tool to Assess Systematic Reviews (AMSTAR‐2) and the Risk of Bias in Systematic Reviews (ROBIS) for critically appraising evidence by pharmacy students.

**Materials and Methods:**

Students attended eight lectures on evidence‐based medicine. Students independently assessed two SRs using AMSTAR‐2 and ROBIS. The agreement between both tools were calculated using Spearman's test while interrater reliability was calculated using Fleiss' *κ* statistics.

**Results:**

Students reported a preference for the AMSTAR‐2 tool due to its clear and distinct rating criteria as well as guidance provided by the tool's developer. In comparison, students found the items on the ROBIS tool difficult to judge as it was subjective. A moderate agreement between both tools on the overall domain ratings was noted (Spearman *r*_s_ = 0.60). There was slight agreement in the overall confidence using AMSTAR‐2 (*κ* = 0.05; 95% confidence interval [CI]: 0.01–0.12) and the overall domain in ROBIS (*κ* = 0.09; 95% CI: 0.01–0.16).

**Conclusion:**

The AMSTAR‐2 tool had a low level of concordance in ratings of review among students. However, the AMSTAR‐2 tool was preferred by students due to the clear guidance and ease of use.

## 1 INTRODUCTION

Evidence‐based medicine (EBM) is taught in medical curricula worldwide in both undergraduate and postgraduate courses. The aim is to provide learners with the ability and skills to make the most appropriate medical decision by integrating the best available evidence, clinical expertise, and patient preference. Systematic reviews (SRs) are often used by healthcare decision‐makers as a reliable, succinct, and credible source of information to achieve evidence‐based healthcare. As such, it is important that healthcare professionals can identify high‐quality SRs that have been conducted following a rigorous process that is fully and transparently reported. As the quality of SRs varies, it is crucial that healthcare students appraise the evidence before decision‐making.

One of the earliest tools used to appraise the methodological quality of SRs was the A MeaSurement Tool to Assess Systematic Reviews (AMSTAR) tool [1]. The tool was developed in 2007, and contains 11 items with four response categories. However, AMSTAR had some limitations since it could not assess the quality of nonrandomized studies and lacked an overall score which prevents comparison across different reviews [2]. In addition, the tool also lacked an item to assess subgroup and sensitivity analysis. In 2017, AMSTAR was refined in an attempt to overcome these weaknesses and was published as the AMSTAR‐2 tool [3]. The new tool contains 16 items with simplified response categories, and includes detailed guidance for reviewers.

Another tool that has undergone rigorous development is the Risk Of Bias In Systematic reviews (ROBIS) tool, which aims to facilitate the appraisal of risk of bias (ROB) in SRs [4]. The tool has three distinct phases. The first phase is an optional phase to assess the applicability of a SR to the research question of interest. The second phase contains four key domains, each with five response options to identify concerns about the review conduct. The third phase consists of three signaling questions which enables an overall assessment of bias rating of either “low,” “high,” or “unclear” risk of bias. ROBIS has been used widely for evaluating bias present within SRs of intervention effectiveness, diagnostic test accuracy, prognosis, and aetiology.

While both tools are intended to be used to assess the quality of a SR, they assess similar yet distinct items. For example, the AMSTAR‐2 tool includes items such as the detailed list of excluded studies in SRs and conflict of interest reporting by trials that are not evaluated using ROBIS, since these are considered issues related to methodological quality. Instead, more emphasis is given to the data synthesis by the ROBIS tool. Several studies have recently compared the reliability and usability of these tools for reviews of both randomized and nonrandomized trials among experienced systematic reviewers or methodologists [5, 6, 7]. These studies have reported a high interrater reliability (IRR) for both tools, albeit a lower IRR value for ROBIS [6]. Meanwhile, another study reported a higher preference for AMSTAR‐2 to rate SRs compared to ROBIS [7].

## 2 OBJECTIVE

To the best of our knowledge, there is limited evidence on which of the utility tools is the most appropriate to teach healthcare students critical appraisal of SRs. The aim of this study was to report our first‐hand experience on students' perception of using the AMSTAR‐2 or ROBIS tool, the validity, reliability, and applicability.

## 3 METHODS

### 3.1 Participants

At the School of Pharmacy in Monash University Malaysia, students are introduced to critical appraisal of literature during their 2nd year of studies. In this 4‐week course, students are trained to formulate a research question, identify the best available information and appraise the clinical question relevant to pharmacy practice. During the course, a series of knowledge‐based interactive lectures focused on EBM concepts, including epidemiological study designs, statistical methods, search strategies, and the step‐by‐step process of conducting a SR is delivered to students (see Table 1). Each lecture is followed by a 2‐h workshop aimed at ensuring students can integrate the knowledge gained to provide them with the practical context to critically appraise health science literature.

**Table 1:** Overview of topics covered in the evidence‐based medicine module.

Topic covered	Subtopic	EBP domain
Evidence and clinical decision making	Translating EBM to practice and asking questions using Patient‐Intervention‐Comparison–Outcome format	Ask, Acquire, Appraise
	Evidence and clinical decision making‐Retrieving evidence and statistical concepts	Appraise
Study designs	Observational study designs	Ask, Appraise
	Prospective study designs	Ask, Appraise
Appraising, critically evaluating, and synthesizing evidence	Interpreting epidemiological studies and sources of bias in studies	Ask, Acquire, Appraise
	Critical appraisal of evidence	Ask, Acquire, Appraise
How evidence informs clinical practice	Systematic reviews and meta‐analysis	Ask, Acquire
	How evidence is incorporated into clinical practice	Ask, Acquire, Appraise. Apply

*Note*: A 2‐h workshop was scheduled 2 days after the delivery of each subtopic to enable students to apply the concepts learned. The total contact time was 8 h, with 4 h of directed self‐study.

However, faculty members teaching the course found that students had difficulty understanding and appraising SRs when asked to apply this knowledge in a mock guideline development panel exercise. This was identified as important feedback and opportunity for further improvement.

### 3.2 Study design

As part of the workshop, students were required to read and critically appraise two SRs. The SRs were chosen based upon the following criteria: (1) met the PRISMA‐P (PRISMA for protocol) definition of a systematic review; (2) included randomized controlled studies only; (3) published in English language; and (4) was listed as a recommended reading for students related to the topic of respiratory or gastrointestinal systems. References were excluded if they described nonsystematic reviews, scoping reviews, or summaries of SRs.

The two topics were chosen since students studied these disease states in their previous semester. The SR would serve to reinforce students learning as a topic refresher. The author retrieved the list of recommended reading from the learning management system and selected potential SRs that could be used for this study. A team of educators comprising of a medical librarian and two faculty members from the Faculty of Pharmacy met up and selected a total of five SRs for the workshop [8, 9, 10, 11, 12]. The reviews were published between 2005 and 2017, and investigated several pharmacological or complementary alternative therapies for the treatment of respiratory or gastrointestinal disease. All but one study [11] included quantitative analyses, and three were Cochrane reviews, since previous studies have indicated that Cochrane reviews have higher methodological and reporting quality [13].

Previous studies have reported that it would take between 10 and 20 min to evaluate a SR using the AMSTAR‐2 or ROBIS tool. As the educational workshop was only 120 min, we took a pragmatic approach and assigned students to evaluate only two SRs during the workshop (Table 2).

**Table 2:** Detailed schedule of workshop and learning objectives.

Learning outcomes	Workshop activity	Type of learning^a^	Duration (min)
After completing the workshop, students will be able to:			
1. Identify and appraise research evidence as the basis for clinical decision making	Instructor‐led class discussion and demonstration on how to appraise SR using the AMSTAR‐2 and ROBIS tool	Knowledge: comprehension	30
	Students hands‐on‐activity of appraising SR using AMSTAR‐2 and ROBIS tool	Knowledge, Attitude and Skills: Comprehension, application, analysis, synthesis, evaluation	60
2. Communicate research evidence in oral and written language appropriate for different clinical contexts	Each subgroup of students discussed the SR and explained to the wider workshop group and instructor on the quality and risk of SR	Knowledge; Skill: Comprehension, analaysis, synthesis, evaluation	30

^a^ Classification of cognitive domain based on Bloom's Taxonomy of Learning.

**Figure 1:**
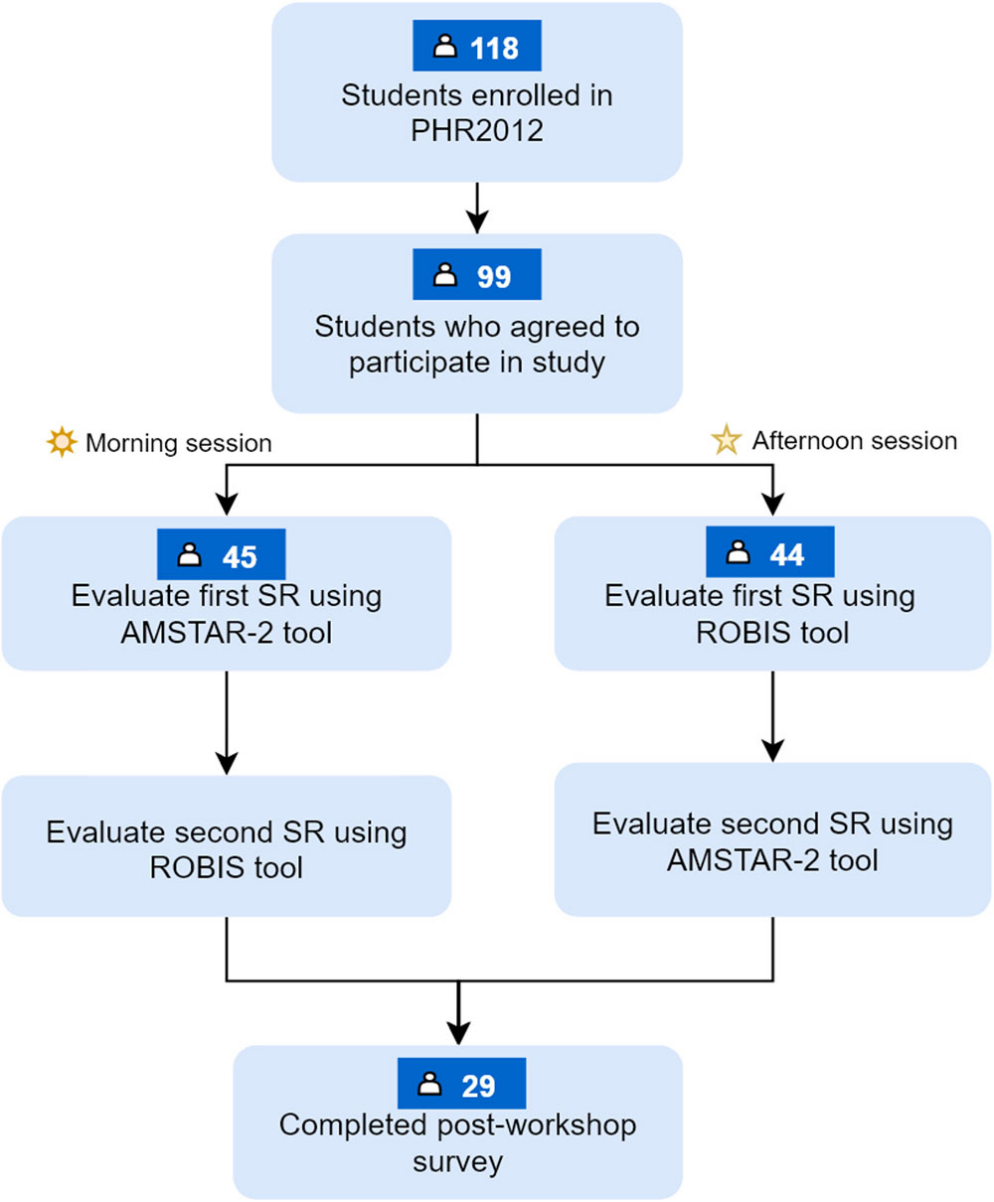
Study flow.

Students were randomly assigned using a computer‐generated permuted block design to evaluate two of the five SRs. These SRs were available on our learning management software Moodle^TM^ 1 week before the workshop. Students were requested to read these reviews beforehand. Due to the large number of students enrolled in this course, students were assigned to attend the workshop scheduled either in the morning or evening (Figure 1). During the workshop, a short introductory lecture (30 min) was given to students on how to navigate and use the AMSTAR‐2 and ROBIS tool. Students who attended the workshop in the morning were asked to appraise the first SR using AMSTAR‐2 tool followed by the second SR using ROBIS tool. For students who attended the workshop in the evening, we switched the sequence and requested students to appraise the first SR using ROBIS tool and second using AMSTAR‐2 tool.

### 3.3 Quality appraisal of SRs

Students were asked to rate the assigned SR as intended by the authors of tools. For the AMSTAR‐2 tool, students assigned a decision of yes or no for items 1, 3, 5, 6, and 10 to 16. For Items 2, 4, 7, 8, and 9, students assigned a decision of yes, partial yes or no while for items 11, 12, and 15, a decision of not applicable was applied. Based upon the decision rules suggested by Shea et al, a rating of high, moderate, low, or critically low for the overall confidence in the results was assigned. For ROBIS tool, students applied a decision of yes, probably yes, probably no, no, or no information to each of the signaling questions within the four risk of bias domains and overall.

After students completed the ratings of the two assigned SRs, 10 students were asked to present their ratings of the assigned reviews to the wider class. Three faculty members (minimum 2 years' experience in EBM training) provided feedback and clarified concepts related to rating SRs if needed. Upon completion of the workshop, students completed an evaluation form to gauge the usability and preference of tools to critically appraise the SRs, challenges encountered, and their perceived confidence in appraising evidence. Students were asked to rank ease of use for the ROBIS and AMSTAR‐2 tools based upon a 7‐point scale (ranging from 1: *extremely difficult* to 7: *extremely easy*); “How difficult (1) or easy (7) was it to use the ROBIS tool?” and “How difficult (1) or easy (7) was it to use the AMSTAR‐2 tool?”. Students also had the opportunity to provide the reasons for their decision (see Supporting Information: Appendix for list of questions). Students were not assessed at baseline to avoid the item‐practice effect which [14]. Responses were collected online using Qualtrics (Qualtrics).

### 3.4 Data analysis

The data were summarized using descriptive statistics as means and standard deviations for continuous variables and frequencies for categorical variables. The confidence in the SR rating for each tool was converted into numerical values to determine the construct validity. Values of 1 for critically low‐quality to 4 for high quality were assigned to SRs rated using AMSTAR‐2, while values of 1 for high risk of bias to 3 for low risk of bias was assigned for SRs rated using the ROBIS tool [5]. The mean score for each SR was used to calculate Spearman's rank correlation coefficient (*r*_s_). No consensus took place since the aim of the current study was to determine the applicability, usability, and preference of the tools for teaching.

In addition, the interrater agreement for all items and their overall rating for both tools were calculated using Fleiss' Kappa (*κ*) statistics with their corresponding 95% confidence intervals (CI). Values of 1 for yes, 2 for partial yes, and 3 for no were assigned for items 2, 4, 7, 8, and 9 of the AMSTAR‐2 tool. For items 11, 12, and 15, a value of 4 was used for the variable “not applicable.” The agreement on rating each item were classified as poor (<0.00), slight (0.01–0.20), fair (0.21–0.40), moderate (0.41–0.60), substantial (0.61–0.80), and almost perfect (0.81–1.00) [15]. The total time taken to complete each tool (excluding reading time for each review) was calculated and presented as mean with corresponding standard deviation (SD) as a measure for tool usability, and compared using a *t*‐test. A comparison was done to determine the duration taken to evaluate Cochrane reviews and non‐Cochrane reviews. All analyses were performed using SPSS Version 27. Ethical approval was obtained from Monash University Human Research Ethics Committee (MUHREC Project: 21293).

## 4 RESULTS

A total of 118 students were invited to participate in this study; 99 agreed and were included. Of these, another 29 students also completed the postworkshop evaluation survey (Figure 1). Students had a mean age of 20.5 (±0.7) years and were mostly females (80%), which was generally representative of the cohort of pharmacy students at our institution.

### 4.1 Tool usability

The mean time needed to complete scoring a SR using the AMSTAR‐2 tool (34.8 min ± 10.6 min) was significantly longer compared to ROBIS (24.9 min ± 12.0 min; *p* < 0.001). We did not find any influence on the type of review (either with meta‐analysis or without) or between Cochrane and non‐Cochrane reviews on the time needed for students to score on the tool (Table 3). Postworkshop questionnaire indicated that 97% of respondents reported a preference to use AMSTAR‐2 tool for critical appraisal of evidence (Supporting Information: Appendix Table 1). Students found the detailed guidance provided by the AMSTAR‐2 tool to be very useful and simple to understand. In comparison, students found that the ROBIS tool was rather ambiguous due to the availability of multiple choices which made it very difficult to judge. They also reported that rating on several questions were very subjective such as item 1.3 which focused on eligibility criteria, and item 2.3 on search comprehensiveness.

**Table 3:** Time taken to evaluate each review based upon tool used.

	Time taken (SD), min	*p* Value
Tool		<0.001
AMSTAR‐2	34.8 (10.6)	
ROBIS	24.9 (12.0)	
AMSTAR‐2 tool		0.38
Cochrane review	36.5 (11.3)	
Non‐Cochrane review	34.2 (11.5)	
ROBIS tool		0.44
Cochrane review	21.9 (2.3)	
Non‐Cochrane review	24.2 (9.6)	
Type of review evaluated		0.28
Systematic review only	29.9 (13.1)	
Systematic review with meta‐analysis	30.0 (14.4)	

### 4.2 Overall quality judgment

**Figure 2:**
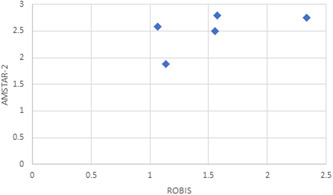
Comparison of mean overall ratings for AMSTAR 2 and ROBIS. Each diamond represents one systematic review.

There was moderate agreement and concordance between students using both tools regarding the overall AMSTAR‐2 confidence in review rating and overall domain rating in ROBIS (*r*_s_ = 0.60, Figure 2). Based on students' judgment, 71.6% reviews were judged to be critically low quality, 12.7% low quality, 5.9% moderate quality, and 9.8% high quality using the AMSTAR‐2 tool. In comparison, 11.8% reviews were judged to have a high risk of bias, 34.8% had unclear risk of bias, and 54.3% had a low risk of bias using the ROBIS tool.

### 4.3 IRR

We found substantial variability in the IRR for all the items on the AMSTAR‐2 tool (Supporting Information: Appendix Table 2). The lowest IRR was observed for items 4, 5, 8, 11, 13, 15, and 16 which indicates minimal agreement among students on these ratings. There was only slight agreement on the overall confidence in the SR (*κ* = 0.05, 95% CI: 0.01–0.12). Similarly, there was slight agreement in students' rating for all domains using the ROBIS tool (*κ* = 0.01–0.08, Supporting Information: Appendix Table 3) and the IRR for the overall domain was poor as indicated by *κ* = 0.09 (95% CI: 0.01–0.16).

## 5 DISCUSSION

Critical appraisal of articles is important to the practice of EBM, as it allows healthcare professionals to be able to draw conclusions regarding the validity and generalizability of a study. However, the best format for teaching critical appraisal is uncertain. Studies have used a wide variety of methods including clinically integrated methods, whereby educational activities were integrated into clinical practice as well as stand‐alone instructions such as the current study [16, 17, 18, 19].

Results suggest that students found the grading of SRs to be challenging and was highly variable using both AMSTAR‐2 and ROBIS. The IRR ratings were considerably low, with poor concordance on the overall ratings between both tools. We offer several explanations for this observation. Firstly, this was an exploratory study to determine the applicability of AMSTAR‐2 and ROBIS for teaching healthcare students' critical appraisal skills. Unlike most other studies that evaluated the tools' applicability using experienced reviewers [5, 20], our study was conducted among students who had minimal experience in conducting SRs. While we are unaware of similar studies that have attempted to compare the usability of these tools among students, a recent publication compared the concordance of these tools among experienced reviewers [5]. In the study by Pieper and colleagues, the authors reported similarly poor IRR ratings among the four reviewers, with a Fleiss' *κ* value of 0.30 (95% CI: 0.17–0.43) for AMSTAR‐2 and 0.28 (95% CI: 0.13–0.42) for ROBIS.

Second, the differences in study design, conduct, and reporting, may have impacted students' ratings resulting in the low IRR observed. For example, in the study by Useem and colleagues, the authors compared a matched pair analysis of Cochrane and non‐Cochrane reviews [21]; these authors found that despite results being topic‐matched, results were discrepant with very low overlap of included studies. Third, students only evaluated two of the five SRs reported in this study which may partially explain the low IRR ratings of our study. Finally, the course duration of 8 h over 4 weeks was likely to be too short to allow for a deeper understanding of critical appraisal of studies. As such, students may not have completely understood how to rate the reviews using the AMSTAR‐2 or ROBIS tool, leading to spurious results. However, given the exploratory nature of this study, we believe that the results may be useful to guide future studies of a similar nature and provide useful insights for teaching.

In our study, students took an average of 25 min to complete the ROBIS tool and 35 min to complete the AMSTAR‐2 tool. In comparison, other studies have reported shorter times of 18–20 min to complete the AMSTAR‐2 tool and 16–29 min for the ROBIS tool [5, 22]. Indeed, our study is the first to report that students took a longer time to complete the AMSTAR‐2 tool compared to the ROBIS tool. We offer several possible explanations for this observation. As this study examined second‐year students who had minimal experience using these tools, the longer time required is not unexpected. Students often had to read the assigned SR repeatedly before assigning any rating to each item on the tool. Nevertheless, open‐ended responses received from students found that despite the longer time taken, most students preferred to use the AMSTAR‐2 tool compared to ROBIS, due to the clear and distinct items on the tool. Students particularly liked the detailed accompanying instructions associated with the AMSTAR‐2 tool.

Although the ROBIS tool does include guidance, the documents are voluminous and require a considerable expertise and familiarity with study designs. It also relies heavily on subjective judgment [5, 20, 22]. This finding was similarly highlighted by students in our study, where they found difficulty in making judgments on several of the questions listed. For example, on the item of appropriateness of eligibility criteria (Q 1.2), students had varying levels of opinions on what would be an appropriate study design and population to answer the research question. Several students required that the review use the PECOT (Population, Exposure, Comparator, Outcome, Time) framework when assessing this question while others only applied the PICO (Population, Intervention, Comparison, Outcome) framework, which may have led to this divergence [23]. Several students also lamented that they lacked the clinical expertise and content knowledge needed to effectively apply and use the tool effectively. This was similarly observed in the AMSTAR‐2 tool, where we noted only fair concordance between students' ratings. Nevertheless, this finding is not unique to this study; a recent study similarly reported a low concordance between experienced reviewers [22, 24]. This suggests that there may be some misinterpretation on several items of the AMSTAR‐2, leading to the differences in overall confidence of results.

To the best of our knowledge, our study was novel as it attempted to examine which tool would be the most appropriate to teach critical appraisal to undergraduate pharmacy students. While the poor level of concordance between student ratings limited our ability to draw firm conclusions, based upon the feedback from students, we will use the AMSTAR‐2 tool in future courses. As part of future iterations of this workshop, we plan to pre‐record a tutorial on how instructors would generally rate a review. We also plan to provide detailed guidance documents, especially for domains that were found to be ambiguous and where most students struggled in rating.

There are limitations that need to be taken into consideration when interpreting the results of our study. Students only evaluated SRs that included randomised controlled trials. We limited the reviews to two disease categories since students had been taught these topics in the current or previous semester. In future work, introducing other clinical topics or study designs (e.g., cohort studies, nonrandomized controlled studies) would be interesting but may make the results more difficult to interpret. As the reviews selected were part of the students' reading list, this may have introduced bias into the SR selection process. Students only evaluated a small number of SRs which may not provide an accurate representation of tool usability and preference.

We also did not perform a qualitative study to determine the usability and perception of students regarding the tools. Few students provided us with additional free‐text response on the tool's usability. A larger study among students from different healthcare professions and educational institutions would likely provide more generalizable results, particularly if students rated a larger number of SRs and included different study designs.

### 5.1 Future research suggestions

An aspect that should be explored in future studies is to determine how the complexity of the topic and completeness of SR reporting might influence the results in terms of IRR, ease of tool use, and time taken to complete. While we did not explicitly examine this in our study, a previous study by Berkman and colleagues found that IRR of grading strength of evidence among experienced systematic reviewers varied greatly, depending on the type of SR assessed [25]. These authors concluded that better guidance is needed, especially for reviewers who are relatively new to the field. The low IRR reported in our analysis may be partly explained by the fact that our study cohort included students who had minimal experience in evaluating SRs.

## 6 CONCLUSIONS

In summary, we found that there was a low level of concordance in the ratings of SRs among students using both AMSTAR‐2 and ROBIS. While the ROBIS tool was completed faster, most student expressed preference for AMSTAR‐2 tool due to the explicit guidance provided and less reliance on subjective judgments.

## AUTHOR CONTRIBUTIONS

**Shaun W. H. Lee**: Conceived study; conducted experiment; data collection and analyses; drafted manuscript; approved final version paper; guarantor of overall content study.

## CONFLICT OF INTEREST STATEMENT

The author declares no conflict of interest.

## Supporting information

Supporting information.

## Data Availability

The data that support the findings of this study are available from the corresponding author upon reasonable request.

## References

[1] Shea BJ , Grimshaw JM , Wells GA , et al. Development of AMSTAR: a measurement tool to assess the methodological quality of systematic reviews. BMC Med Res Methodol. 2007;7(1):10. 17302989 10.1186/1471-2288-7-10 PMC1810543

[2] Burda BU , Holmer HK , Norris SL . Limitations of a measurement tool to assess systematic reviews (AMSTAR) and suggestions for improvement. Syst Rev. 2016;5:58. 10.1186/s13643-016-0237-1 27072548 PMC4830078

[3] Shea BJ , Reeves BC , Wells G , et al. AMSTAR 2: a critical appraisal tool for systematic reviews that include randomised or nonrandomised studies of healthcare interventions, or both. BMJ. 2017;358:j4008. 28935701 10.1136/bmj.j4008 PMC5833365

[4] Whiting P , Savović J , Higgins JPT , et al. ROBIS: a new tool to assess risk of bias in systematic reviews was developed. JCE. 2016;69:225‐234. 26092286 10.1016/j.jclinepi.2015.06.005 PMC4687950

[5] Pieper D , Puljak L , González‐Lorenzo M , Minozzi S . Minor differences were found between AMSTAR 2 and ROBIS in the assessment of systematic reviews including both randomized and nonrandomized studies. JCE. 2019;108:26‐33. 10.1016/j.jclinepi.2018.12.004 30543911

[6] Lorenz RC , Matthias K , Pieper D , et al. A psychometric study found AMSTAR 2 to be a valid and moderately reliable appraisal tool. JCE. 2019;114:133‐140. 31152864 10.1016/j.jclinepi.2019.05.028

[7] Perry R , Whitmarsh A , Leach V , Davies P . A comparison of two assessment tools used in overviews of systematic reviews: ROBIS versus AMSTAR‐2. Syst Rev. 2021;10(1):273. 10.1186/s13643-021-01819-x 34696810 PMC8543959

[8] Gill SK , O'Brien L , Einarson TR , Koren G . The safety of proton pump inhibitors (PPIs) in pregnancy: a meta‐analysis. Am J Gastroenterol. 2009;104(6):1541‐1545. 10.1038/ajg.2009.122 19491869

[9] Horita N , Goto A , Shibata Y , et al. Long‐acting muscarinic antagonist (LAMA) plus long‐acting beta‐agonist (LABA) versus LABA plus inhaled corticosteroid (ICS) for stable chronic obstructive pulmonary disease (COPD). Cochrane Database Syst Rev. 2017;2018(2):Cd012066. 10.1002/14651858.CD012066.pub2 PMC6464543 28185242

[10] Jefferson T , Jones MA , Doshi P , et al. Neuraminidase inhibitors for preventing and treating influenza in adults and children. Cochrane Database Syst Rev. 2014;2018(4):Cd008965. 10.1002/14651858.CD008965.pub4 PMC6464969 24718923

[11] Ramkumar D , Rao SSC . Efficacy and safety of traditional medical therapies for chronic constipation: systematic review. Am J Gastroenterol. 2005;100(4):936‐971. 10.1111/j.1572-0241.2005.40925.x 15784043

[12] Karsch‐Völk M , Barrett B , Kiefer D , Bauer R , Ardjomand‐Woelkart K , Linde K . Echinacea for preventing and treating the common cold. Cochrane Database Syst Rev. 2014;2014(2):Cd000530. 10.1002/14651858.CD000530.pub3 24554461 PMC4068831

[13] Petticrew M , Wilson P , Wright K , Song F . Quality of Cochrane reviews: quality of Cochrane reviews is better than that of non‐Cochrane reviews. BMJ: British Medical Journal. 2002;324(7336):545. 10.1136/bmj.324.7336.545/a PMC1122457 11872564

[14] Welch WW , Walberg HJ . Pretest and sensitization effects in curriculum evaluation. Am Educ Res J. 1970;7(4):605‐614.

[15] Landis JR , Koch GG . The measurement of observer agreement for categorical data. Biometrics. 1977;33(1):159‐174. 10.2307/2529310 843571

[16] Ahmadi SF , Baradaran HR , Ahmadi E . Effectiveness of teaching evidence‐based medicine to undergraduate medical students: a BEME systematic review. Med Teach. 2015;37(1):21‐30. 10.3109/0142159x.2014.971724 25401408

[17] Lehane E , Leahy‐Warren P , O'Riordan C , et al. Evidence‐based practice education for healthcare professions: an expert view. BMJ Evid Based Med. 2019;24(3):103‐108. 10.1136/bmjebm-2018-111019 PMC6582731 30442711

[18] Zeleníková R , Beach M , Ren D , Wolff E , Sherwood P . Faculty perception of the effectiveness of EBP courses for graduate nursing students. Worldviews Evid Based Nurs. 2014;11(6):401‐413. 10.1111/wvn.12068 25270089

[19] Larsen CM , Terkelsen AS , Carlsen A‐MF , Kristensen HK . Methods for teaching evidence‐based practice: a scoping review. BMC Med Educ. 2019;19(1):259. 10.1186/s12909-019-1681-0 31296212 PMC6624945

[20] Banzi R , Cinquini M , Gonzalez‐Lorenzo M , Pecoraro V , Capobussi M , Minozzi S . Quality assessment versus risk of bias in systematic reviews: AMSTAR and ROBIS had similar reliability but differed in their construct and applicability. JCE. 2018;99:24‐32. 29526556 10.1016/j.jclinepi.2018.02.024

[21] Useem J , Brennan A , LaValley M , et al. Systematic differences between Cochrane and non‐Cochrane Meta‐Analyses on the same topic: a matched pair analysis. PLoS One. 2015;10(12):e0144980. 10.1371/journal.pone.0144980 26671213 PMC4686011

[22] Gates M , Gates A , Duarte G , et al. Quality and risk of bias appraisals of systematic reviews are inconsistent across reviewers and centers. JCE. 2020;125:9‐15. 10.1016/j.jclinepi.2020.04.026 32416337

[23] Schardt C , Adams MB , Owens T , Keitz S , Fontelo P . Utilization of the PICO framework to improve searching PubMed for clinical questions. BMC Med Inform Decis Mak. 2007;7:16. 10.1186/1472-6947-7-16 17573961 PMC1904193

[24] Pieper D , Lorenz RC , Rombey T , et al. Authors should clearly report how they derived the overall rating when applying AMSTAR 2: a cross‐sectional study. JCE. 2021;129:97‐103. 10.1016/j.jclinepi.2020.09.046 33049325

[25] Berkman ND , Lohr KN , Morgan LC , Kuo T‐M , Morton SC . Interrater reliability of grading strength of evidence varies with the complexity of the evidence in systematic reviews. JCE. 2013;66(10):1105‐1117.e1. 10.1016/j.jclinepi.2013.06.002 23993312

