# Supplementary material for: What tool do undergraduate pharmacy students prefer when grading systematic review evidence: AMSTAR‐2 or ROBIS?
Source: Cochrane Evid Synth Methods. 2023 Aug 9;1(6):e12023. doi: 10.1002/cesm.12023 (PMC11795889; doi:10.1002/cesm.12023)
Supplement: Supplementary file 1 — Supporting information. [file CESM-1-e12023-s001.docx]

Appendix Table 1: Time taken to evaluate each review based upon tool used

|  | **Time taken (SD), mins** | **p-value** |
| --- | --- | --- |
| **Tool** |  | <0.001 |
| AMSTAR-2 | 34.8 (10.6) |  |
| ROBIS | 24.9 (12.0) |  |
| **AMSTAR-2 tool** |  | 0.38 |
| Cochrane review | 36.5 (11.3) |  |
| Non-Cochrane review | 34.2 (11.5) |  |
| **ROBIS tool** |  | 0.44 |
| Cochrane review | 21.9 (2.3) |  |
| Non-Cochrane review | 24.2 (9.6) |  |
| **Type of review evaluated** |  | 0.28 |
| Systematic review only | 29.9 (13.1) |  |
| Systematic review with meta-analysis | 30.0 (14.4) |  |

Appendix Table 2: Student’s ranking of preference on tool for learning

| **Variable** | **N (%)** |
| --- | --- |
| Gender, n (%) |  |
| Female | 24 (82.8) |
| Male | 5 (17.2) |
| Please rank your preference for tool to be used for rating a quality of systematic review, n (%) |  |
| AMSTAR-2 | 28 (97) |
| ROBIS | 1 (3) |
| Please rate the ease of use for each of the following tool (mean, SD)* |  |
| AMSTAR-2 | 3.0 (1.26) |
| ROBIS | 2.3 (1.06) |

*Measured using a 7-point rating scale (1: extremely difficult to 7: extremely easy)
